# The Effect of Alloying Elements on the Structural Stability, Mechanical Properties, and Debye Temperature of Al_3_Li: A First-Principles Study

**DOI:** 10.3390/ma11081471

**Published:** 2018-08-18

**Authors:** Jinzhong Tian, Yuhong Zhao, Hua Hou, Bing Wang

**Affiliations:** School of Materials Science and Engineering, North University of China, Taiyuan 030051, China; tjz028@163.com (J.T.); houhua@nuc.edu.cn (H.H.); nicebing1135@163.com (B.W.)

**Keywords:** first-principles, doping concentration, alloying elements, mechanical properties, Debye temperature, lightweight structural materials, Al_3_Li

## Abstract

The structural stability, mechanical properties, and Debye temperature of alloying elements X (X = Sc, Ti, Co, Cu, Zn, Zr, Nb, and Mo) doped Al_3_Li were systematically investigated by first-principles methods. A negative enthalpy of formation Δ*H_f_* is predicted for all Al_3_Li doped species which has consequences for its structural stability. The Sc, Ti, Zr, Nb, and Mo are preferentially occupying the Li sites in Al_3_Li while the Co, Cu, and Zn prefer to occupy the Al sites. The Al–Li–X systems are mechanically stable at 0 K as elastic constants *C*_ij_ has satisfied the stability criteria. The values of bulk modulus *B* for Al–Li–X (X = Sc, Ti, Co, Cu, Zr, Nb, and Mo) alloys (excluding Al–Li–Zn) increase with the increase of doping concentration and are larger than that for pure Al_3_Li. The Al_6_LiSc has the highest shear modulus *G* and Young’s modulus *E* which indicates that it has stronger shear deformation resistance and stiffness. The predicted universal anisotropy index *A^U^* for pure and doped Al_3_Li is higher than 0, implying the anisotropy of Al–Li–X alloy. The Debye temperature *Θ_D_* of Al_12_Li_3_Ti is highest among the Al–Li–X system which predicts the existence of strong covalent bonds and thermal conductivity compared to that of other systems.

## 1. Introduction

Lightweight structural materials such as the Al–Li based alloys have excellent comprehensive performance, such as low density, good corrosion resistance, and high elastic modulus [1,2], and it is the basic reason why Al–Li based alloys are so widely used in aviation and aerospace field. The metastable Al_3_Li (δ′) precipitates has an important influence on the mechanical properties of Al–Li based alloys [3,4]. The δ′ phase is highly ordered with an L1_2_ structure and forms as spheres possessing a cube–cube orientation relationship with matrix [5]. The lattice constant of δ′ phase (4.02 Å) and dilute Al–Li solid solutions (4.04 Å) are almost equal, and the corresponding precipitate-to-matrix misfit results in an interfacial strain of approximately 0.08 ± 0.02% [6]. Due to the small lattice misfit, strong orientational habit and low interfacial strains, the δ′ phase remain crystallographically coherent with the parent solid-solution matrix and the crystallographic orientation relationship is (111)_Al3Li_//(111)_Al_ [7,8]. The δ′ precipitates are considered the most important strengthening phases of Al–Li alloys [9].

As far as the importance of δ′ precipitates is concerned, its structure and electronic properties, mechanical properties, nucleation and growth mechanism, and coarsening behavior have been widely studied both experimentally and theoretically [10,11,12,13]. The solubility and stability of δ′ phase in A1–Li alloy have been reported by Mao et al. [14], which suggest that vibrational entropy is essential for the simulation of solubility. Phase-field method is applied for the investigation of coarsening kinetics of δ′ precipitates in the binary Al–Li alloys [15]. The formation enthalpy, electronic structures, and vibrational and thermodynamic properties of the δ′ phase were systematically reported by employing the first-principles methods [16,17,18,19,20,21]. Yao et al. have reported that point defects play an important role in determining the physical properties of off-stoichiometric δ′ phase [22]. The δ′ phase in binary Al–Li alloys provide limited room temperature strength due to their inability to form in high volume fractions unlike precipitates in Al–Cu or Al–Zn–Mg alloys [23]. In this regard, it is important to improve the mechanical properties of δ′ phase. For strengthening phases, doping with the additional alloying elements is an effective way to improve their specific properties [24,25,26]. The alloying elements X (X = Sc, Ti, Co, Cu, Zn, Zr, Nb, and Mo) are often adopted to improve the specific properties of alloys. However, no one has reported the influences of alloying elements X (X = Sc, Ti, Co, Cu, Zn, Zr, Nb, and Mo) on the mechanical properties of δ′ phase.

In this paper, first-principles methods were employed to study the effect of alloying elements X (X = Sc, Ti, Co, Cu, Zn, Zr, Nb, and Mo) and doping concentration on the structural stability, elastic properties, hardness, elastic anisotropy, and Debye temperature of Al_3_Li phase.

## 2. Computational Studies

**Figure 1:**
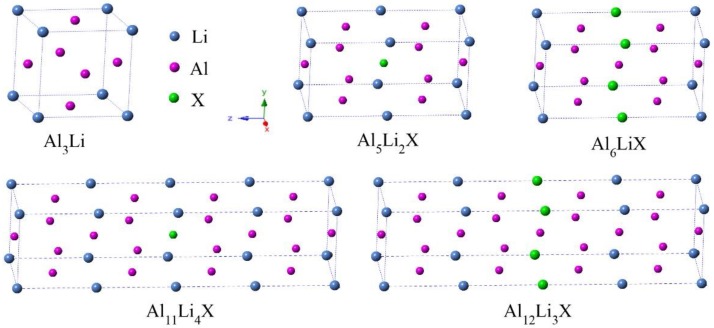
Crystalline structures of Al_3_Li doped with alloying element X (X = Sc, Ti, Co, Cu, Zn, Zr, Nb, and Mo) at different alloying concentrations of 6.25 and 12.5%.

The Al_3_Li phase has a cubic structure with a space group of pm-3m (No. 221), which contains 3 Al atoms and 1 Li atom (see Figure 1). The Al and Li atom occupy 3c (0 0.5 0.5) and 1a (0 0 0) Wyckoff position, respectively. Based on the Al_3_Li phase, the supercells of 1 × 1 × 4 and 1 × 1 × 2 were constructed to study the doping effects at various alloying concentrations of 6.25 and 12.5%, respectively. In the supercells, Al or Li sites can be substituted by single alloying element X (X = Sc, Ti, Co, Cu, Zn, Zr, Nb, and Mo), while the chemical formulas of doped species can be represented as Al_3_Li, Al_6_LiX, Al_5_Li_2_X, Al_12_Li_3_X, and Al_11_Li_4_X, respectively.

All the first-principles calculations were carried out with CASTEP package [27] based on the density functional theory (DFT) [28]. The ultrasoft pseudopotential [29] in reciprocal space was performed to describe the ion-electron interactions. The generalized gradient approximation (GGA) with the Perdew–Burke–Ernzerhof (PBE) function [30] was applied to describe the exchange-correlation potential. Al 3s^2^3p^1^, Li 1s^2^2s^1^, Sc 3s^2^3p^6^3d^1^4s^2^, Ti 3s^2^3p^6^3d^2^4s^2^, Co 3d^7^4s^2^, Cu 3d^10^4s^1^, Zn 3d^10^4s^2^, Zr 4s^2^4p^6^4d^2^5s^2^, Nb 4s^2^4p^6^4d^4^5s^1^, and Mo 4s^2^4p^6^4d^5^5s^1^ were treated as valence electrons. The plane-wave energy cutoff of 500 eV was selected for all calculations. The 21 × 21 × 21 k-points mesh was set for Al_3_Li, and 21 × 21 × 11 and 21 × 21 × 5 k-points meshes were adopted for sampling the 1 × 1 × 4 and 1 × 1 × 2 supercells of Al_3_Li, respectively. The convergence threshold of 5.0 × 10^−6^ eV/atom was chosen for maximum energy change.

## 3. Results and Discussion

### 3.1. Site Preference and Phase Stability

The predicted lattice constants, volume, mass density, and formation enthalpy of pure Al_3_Li phase are listed in Table 1. The DFT is one of the calculation methods to simplify the solution of the Schrodinger equation [31]. Thus, the discrepancies between the calculated and experimental values are inevitable. In Table 1, the obtained lattice constant and mass density is consistent with the experimental values, illustrating the reliability of the present computational model.

The structural stability of doped Al_3_Li phase can be predicted by the simulated value of enthalpy of formation (Δ*H_f_*). The Δ*H_f_* can be calculated with the help of Formula (1) [33].

(1) $$\Delta H_{f}=\frac{1}{n}(E_{\mathrm{tot}}-aE_{\mathrm{solid}}^{\mathrm{Al}}-bE_{\mathrm{solid}}^{\mathrm{Li}}-E_{\mathrm{solid}}^{X})$$

where *E*_tot_ represents the total energy of doped Al_3_Li phase, $E_{\mathrm{solid}}^{\mathrm{Al}}$, $E_{\mathrm{solid}}^{\mathrm{Li}}$, and $E_{\mathrm{solid}}^{X}$ denote the energies per atom of Al, Li, and X in solid states, *n* stands for the total number of atoms in the Al–Li–X system while *a* and *b* are the number of Al and Li atoms. The predicted Δ*H_f_* and site occupancy behaviors of doping elements X (X = Sc, Ti, Co, Cu, Zn, Zr, Nb, and Mo) in Al_3_Li are listed in Table 2. A negative Δ*H_f_* is predicted for all doped Al_3_Li, indicating its structural stability. Generally, a higher negative value of the Δ*H_f_* means the material is more stable. Thus, the Sc, Ti, Zr, Nb, and Mo are preferentially occupying the Li sites in Al_3_Li while the Co, Cu, and Zn prefer to occupy the Al sites. Moreover, the Δ*H_f_* of Al_6_LiZr phase is smaller than other systems, which led us to predict that the occupancy of alloying elements Zr in Li site can substantially improve the stability of alloy.

### 3.2. Mechanical Properties

Elastic constants (*C*_ij_) is an important parameter which can be used to predict the physical properties and mechanical stability of materials [34,35]. In this paper, the *C*_ij_ is obtained by employing the strain–stress method, based on the general Hooke’s law [36,37]. The pure cubic crystal of Al_3_Li has three independent elastic constants, i.e., *C*_11_, *C*_12_, and *C*_44_. As shown in Figure 1, the doped Al_3_Li has tetragonal structure, which is slightly distorted from the pure Al_3_Li crystal [24,38]. Hence, there are six independent elastic constants (*C*_11_, *C*_12_, *C*_13_, *C*_33_, *C*_44_, and *C*_66_) for doped Al_3_Li. Moreover, *C*_ij_ can be used as an important criterion to judge the mechanical stability of pure and doped Al_3_Li. For the cubic crystals this criterion can be used as:

*C*_11_ − *C*_12_ > 0, *C*_11_ > 0, *C*_44_ > 0, *C*_11_ + 2*C*_12_ > 0

For tetragonal crystals:

$$C_{11}>|C_{12}|,\;C_{44}>0,\;C_{66}>0,\;C_{33}(C_{11}+C_{12})>2C_{13}^{2}$$

The predicted *C*_ij_ for pure and doped Al_3_Li at 0 K are summarized in Table 3. The simulated *C*_ij_ of pure Al_3_Li has a strong correlation with the already reported experimental and calculated results [17,39], implying the accuracy of our simulations. As shown in Table 3, all the predicted *C*_ij_ satisfied the stability criteria, indicating that the pure and doped Al_3_Li are mechanically stable at 0 K. All the *C*_ij_ of Al–Li–X (X =Sc, Ti, Zr, and Nb) systems are higher than those of pure Al_3_Li, which indicate that the doping elements X (X =Sc, Ti, Zr, and Nb) can effectively improve the *C*_ij_ of pure Al_3_Li. At high concentration (12.5 at %), the compression for Al_6_LiX (X = Sc, Zr, and Nb) and Al_5_Li_2_X (X = Co, Cu, and Zn) systems in the direction of the x-axis was more difficult than other axis owing the largest *C*_11_, and the deformation resistance for Al–Li–X (X =Ti and Mo) systems along *z*-axis are higher than others due to higher *C*_33_. When the doping concentration drops to 6.25 at %, the *C*_33_ for Al_12_Li_3_X (X = Ti, Zr, Nb, and Mo) systems are higher than other *C*_ij_ demonstrating that *z*-axis exhibits incompressibility.

**Figure 2:**
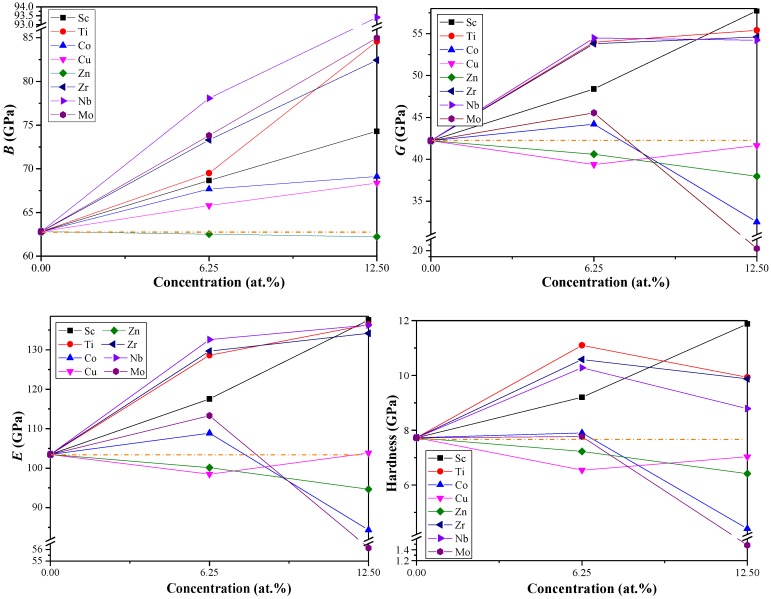
The predicted elastic moduli *B*, *G*, and *E* (GPa) and hardness *H* of Al_3_Li doped with alloying element X (X = Sc, Ti, Co, Cu, Zn, Zr, Nb, and Mo).

The elastic moduli (bulk modulus *B*, shear modulus *G*, and Young’s modulus *E*) of pure and doped Al_3_Li were estimated by Voigt–Reuss–Hill method [40,41]. Generally, *B* describes the resistance to volume change. As shown in Table 3 and Figure 2, the values of *B* for Al–Li–X (X = Sc, Ti, Co, Cu, Zr, Nb, and Mo) alloys increase with the increase of doping concentration which is higher than that of pure Al_3_Li. The greater *B* is, the better the ability to resist to volume change is. When the doping concentration is constant, the values of *B* for Al–Li–Nb are greater than other counterpart species, indicating that Al–Li–Nb has the stronger resistance to volume change. Thus, we inferred that the addition of Nb can effectively improve the resistance to volume change in Al–Li–X. The *G* and *E* measure the resistance to shape change and stiffness of Al–Li–X system, respectively. At low concentration (6.25 at %), the addition of Cu and Zn elements can reduce the *G* and *E* of Al_3_Li. On the other hand, the Al_12_Li_3_Nb has higher values of *G* and *E*. when the doping concentration up to 12.5 at %, the Sc, Ti, Zr, and Nb elements can play an important role in enhancing the *G* and *E* of Al_3_Li. The Al_6_LiSc has higher *G* and *E* which consequently gives stronger shear deformation resistance and stiffness. Comparative analysis of these parameters led us to conclude that the values of *E* (*G*) decrease with the higher concentration (from 6.25 to 12.5%) of Co, Zn, and Mo (Co, Zn, Nb, and Mo). In short, this high doping concentration may decrease the overall performance of the material.

Hardness (*H*) is an important parameter of materials which can be estimated by the ability to resist localized deformation [42]. The defects (i.e., dislocations) and grain sizes of materials have great influence on the hardness [43] and it is very difficult to get the exact value of *H* through empirical method. In this paper, the *H* was roughly predicted by the following semi-empirical formulas [44]:

(2) $$H=\frac{\left(1-2\nu \right)}{6(1+\nu )}E$$

As described in Figure 2, the *H* of Al–Li–Zn follow a descending trend with the increase of doping concentration, but it increases in case of Al-Li-Sc. For Al–Li–X (X = Ti, Co, Zr, Nb, and Mo), the *H* reached to the maximum when 6.25% doping concentration is considered. Besides, the Al_6_LiSc and Al_6_LiMo have maximum and minimum values of *H*, respectively. However, the difference between the hardness of Al_6_LiSc and Al_12_Li_3_Ti is small (about 0.78 GPa). Hence, considering high cost of Sc, the addition of Ti may be the best choice to improve the hardness of pure Al_3_Li.

**Figure 3:**
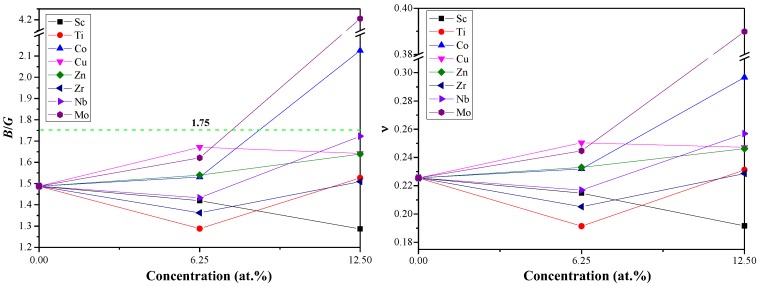
Simulated *B*/*G* and ν of Al_3_Li doped with alloying element X (X = Sc, Ti, Co, Cu, Zn, Zr, Nb, and Mo) as a function of doping concentration.

The brittle or ductile behavior of Al–Li–X system can be roughly evaluated from the ratio of *B*/*G*. Hence, the materials tend to brittle (ductile) if the ratios of *B*/*G* is smaller (larger) than 1.75 [45]. As shown in Figure 3, all the Al–Li–X systems present brittle behavior with a doping concentration of 0 to 6.25 at.%. The Al–Li–X (X = Co and Mo) systems tend to be ductile due to the higher *B*/*G* ratios while other Al–Li–X systems still possess brittle behavior at high concentration (12.5 at.%). Comparative analysis of this behavior led us to suggest that the existence of Co and Mo can transform the intrinsic brittleness of Al_3_Li into ductility. Moreover, the *B*/*G* ratios for the Al–Li–Sc system decrease with the increase of Sc concentration (from 0 to 12.5%), while an increasing trend of *B*/*G* ratios is found in the Al–Li–X (X = Co, Zn, and Mo) systems. In short, it is necessary to choose an appropriate doping element and its concentration for the desired ductility or brittleness of materials.

The Poisson’s ratio ν is defined as *ν* = (3B − 2G)/(6B + 2G) and adopted to reveal the stability of the crystal against shear stress. As shown in Figure 3, the values of ν for Al_6_LiMo and Al_5_Li_2_Co are bigger than those of other Al–Li–X alloys, indicating that Al_6_LiMo and Al_5_Li_2_Co have a higher structural plasticity [46,47]. Besides, the typical values of ν for ionic and metallic materials are 0.25 and 0.33, respectively [48,49]. The predicted ν for Al–Li–X systems (excluding Al_5_Li_2_Co and Al_6_LiMo) are close to 0.25, which shows that main chemical bonding is ionic bonding. The calculated ν for Al_6_LiMo (0.39) and Al_5_Li_2_Co (0.30) are closer to 0.33 than 0.25, implying that the metallic bonding plays the dominant position. Thus, the main chemical bonds of Al_6_LiMo and Al_5_Li_2_Co are different from that of other Al–Li–X alloys, and this may be the reason why Al_6_LiMo and Al_5_Li_2_Co tend to be ductile.

Furthermore, the elastic anisotropy plays a vital role in the mechanical/physical processes such as crack behavior and phase transformations [50]. The elastic anisotropy of pure and doped Al_3_Li can be predicted from the universal anisotropy index (*A^U^*) and its formula is defined as follows [51]:

(3) $$A^{U}=5\frac{G_{V}}{G_{R}}+\frac{B_{V}}{B_{R}}-6$$

where *G_V_* and *G_R_* represent the Voigt and Reuss shear modulus, *B_V_* and *B_R_* are the Voigt and Reuss bulk modulus, respectively.

As listed in Table 3, the predicted *A^U^* for pure and doped Al_3_Li is higher than 0, implying the anisotropy of the Al–Li–X alloy. In case of Al–Li–X alloy, the *A^U^* shows an upward trend with the increase of doping concentration (6.25 to 12.5%). The *A^U^* for most of the Al–Li–X (except Al_5_Li_2_Co, Al_6_LiMo, and Al_12_Li_3_Mo) systems are near zero and smaller than that for pure Al_3_Li, which indicates that most doping elements can reduce the anisotropy of materials. The *A^U^* of Al_6_LiMo is much larger than other species. The reason behind this is that a large difference between *C*_44_ (*C*_11_) and *C*_66_ (*C*_33_) in Al_6_LiMo alloy [52].

### 3.3. Debye Temperature

The Debye temperature *Θ_D_* is an important parameter of a solid and it is associated with thermodynamic properties of materials, such as entropy, thermal expansion, and vibrational internal energy. One of the standard methods of calculating the Debye temperature is from elastic constant data. Thus, the *Θ_D_* was predicted from averaged sound velocity by employing the following formula [53]:

(4) $$\Theta_{D}=\frac{h}{k_{B}}{\left[\frac{3n}{4\pi }(\frac{N_{A}\rho }{M})\right]}^{1/3}v_{m}$$

(5) $$v_{m}={\left[\frac{1}{3}(\frac{2}{{v_{s}}^{3}}+\frac{1}{{v_{l}}^{3}})\right]}^{-1/3}$$

(6) $$v_{s}=\sqrt{\frac{G}{\rho }}$$

(7) $$v_{l}=\sqrt{\frac{3B+4G}{3\rho }}$$

where *h*, *k_B_*, *n*, *N_A_*, *ρ*, *M*, and *v*_m_ stand for Planck’s constant, Boltzmann’s constant, total number of atoms, Avogadro’s number, density, molecular weight, and average wave velocity, respectively. *v_s_* and *v_l_* represent the shear and longitudinal sound velocities of materials, respectively.

**Figure 4:**
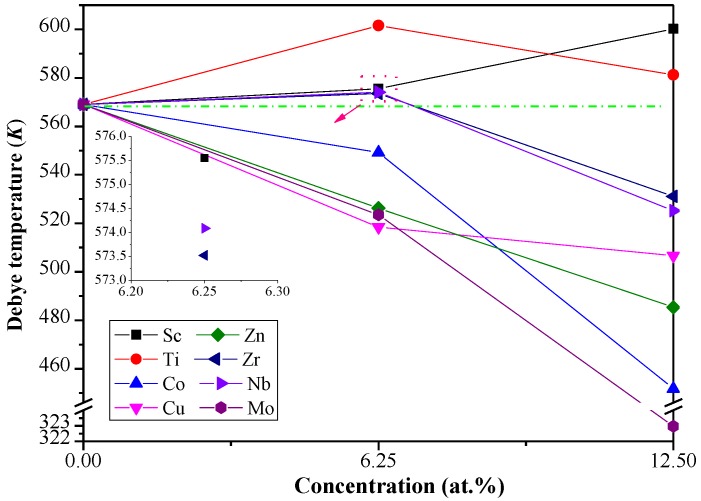
The calculated *Θ_D_* for Al_3_Li doped with alloying element X (X= Sc, Ti, Co, Cu, Zn, Zr, Nb, and Mo) with different doping concentration.

As depicted in Figure 4, the predicted value of *Θ_D_* for pure Al_3_Li is 568.9 K at 0 K, which is in consistent with the already reported data (573 K) [19]. The *Θ_D_* decreases (increases) with the increase of Co, Cu, Zn, and Mo (Sc) concentration in the Al–Li–X systems. In general, a higher *Θ_D_* implies that the materials have higher thermal conductivity and stronger covalent bonds. The *Θ_D_* of Al_12_Li_3_Ti is higher among the Al–Li–X systems, which illustrates that the strength of covalent bonds and thermal conductivity of Al_12_Li_3_Ti are better than others. Furthermore, the predicted density of Al_12_Li_3_Ti (2.5 g/cm^3^) is close to the density of Al_3_Li (2.2 g/cm^3^), which demonstrates thatAl_12_Li_3_Ti may be a better reinforcement phase in aerospace materials. 

## 4. Conclusions

The effect of alloying elements X (X = Sc, Ti, Co, Cu, Zn, Zr, Nb, and Mo) and doping concentration on the structural stability, mechanical properties, and Debye temperature of Al_3_Li were systematically investigated through density functional theory. The main contents of this work can be summarized as follows:

(1) All doped Al_3_Li systems are structural stability. The Sc, Ti, Zr, Nb, and Mo preferentially occupied the Li sites in Al_3_Li while the Co, Cu, and Zn prefer to occupy the Al sites rather than Li sites.

(2) All the *C*_ij_ of Al–Li–X (X = Sc, Ti, Zr, and Nb) systems are higher than those of pure Al_3_Li, which indicate that the doping elements X (X = Sc, Ti, Zr, and Nb) can effectively improve the *C*_ij_ of pure Al_3_Li. The values of *B* for Al–Li–X (X = Sc, Ti, Co, Cu, Zr, Nb, and Mo) alloys (excluding Al-Li-Zn) increase with the increase of doping concentration and are higher than that for pure Al_3_Li. The Al_6_LiSc has higher *G* and *E*, which consequences stronger shear deformation resistance and stiffness.

(3) All the Al–Li–X systems present brittle behavior with the increase in doping concentration (from 0 to 6.25 at %). The Al–Li–X (X = Co and Mo) systems tend to ductile while other Al–Li–X systems still possess brittle behavior at high concentration (12.5 at %), which suggests that the existence of Co and Mo can transform the intrinsic brittleness of Al_3_Li into ductility. Moreover, the Al_6_LiSc and Al_6_LiMo have maximum values of *H* and *A^U^*, respectively.

(4) A higher *Θ_D_* is observed for Al_12_Li_3_Ti, responsible for strong covalent bonds and higher thermal conductivity, compared to other Al–Li–X systems.

## Figures and Tables

**Table 1:** Simulated lattice constant *a* (Å), volume *V* (Å^3^), mass density (Kg/m^3^), and formation enthalpy *H_form_* (eV) of pure Al_3_Li phase at 0 K.

Species	*a*	*V*	Mass Density	*H_form_*
Present	4.034	65.65	2.223	−0.097
Cal. [17]	4.030	65.45	2.221	-
Cal. [14]	4.029	65.40	-	−0.100
Exp. [32]	4.01	64.48	2.260	-

**Table 2:** Simulated enthalpy of formation Δ*H_f_* (eV) and site occupancy behaviors of doping elements X (X = Sc, Ti, Co, Cu, Zn, Zr, Nb, and Mo) in Al_3_Li at 0 K.

Element X	Δ*H_f_*		Site Preference	Element X	Δ*H_f_*		Site Preference
	Al_6_LiX	Al_5_Li_2_X			Al_12_Li_3_X	Al_11_Li_4_X	
Sc	−0.267	−0.154	Li	Sc	−0.180	−0.127	Li
Ti	−0.232	−0.127	Li	Ti	−0.169	−0.108	Li
Co	−0.185	−0.202	Al	Co	−0.140	−0.152	Al
Cu	−0.057	−0.128	Al	Cu	−0.075	−0.111	Al
Zn	−0.017	−0.099	Al	Zn	−0.057	−0.098	Al
Zr	−0.276	−0.148	Li	Zr	−0.191	−0.118	Li
Nb	−0.188	−0.098	Li	Nb	−0.150	−0.094	Li
Mo	−0.100	−0.059	Li	Mo	−0.107	−0.075	Li

**Table 3:** Simulated elastic constants *C*_ij_ (GPa), elastic moduli *B*, *G*, and *E* (GPa), hardness *H* and universal anisotropy index *A^U^* for pure and doped Al_3_Li at 0 K and 0 GPa.

Phase	Species	*C*_11_	*C*_33_	*C*_44_	*C*_66_	*C*_12_	*C*_13_	*B*	*G*	*E*	*H*	*A^U^*
Al_3_Li	Present	129.7	-	37.7	-	29.4	-	62.8	42.2	103.5	7.73	0.099
	Cal. [17]	128	-	39	-	30	-	63.3	42.8	116.8	-	-
	Exp. [39]	123.6	-	42.8	-	37.2	-	66	43	105.9	-	-
Al_12_Li_3_Sc	Present	136.2	130.4	46.1	51.7	31.0	38.3	68.7	48.4	117.6	9.20	0.019
Al_12_Li_3_Ti	Present	141.3	147.7	52.4	54.4	35.2	31.2	69.5	54.0	128.6	11.10	0.007
Al_11_Li_4_Co	Present	138.0	118.9	39.4	48.1	29.0	39.2	67.7	44.2	108.9	7.90	0.085
Al_11_Li_4_Cu	Present	126.7	116.2	37.0	37.5	37.7	37.0	65.8	39.4	98.5	6.55	0.032
Al_11_Li_4_Zn	Present	124.3	121.8	36.9	38.9	33.8	31.2	62.5	40.6	100.1	7.23	0.049
Al_12_Li_3_Zr	Present	143.4	147.5	53.1	55.9	42.2	35.2	73.3	53.8	129.7	10.58	0.008
Al_12_Li_3_Nb	Present	147.8	163.5	53.1	54.9	50.8	35.5	78.1	54.5	132.6	10.28	0.041
Al_12_Li_3_Mo	Present	118.7	147.0	52.0	51.5	66.9	36.6	73.8	45.4	113.3	7.77	0.426
Al_6_LiSc	Present	146.6	136.8	61.3	62.0	35.2	41.3	74.3	57.7	137.6	11.88	0.040
Al_6_LiTi	Present	153.7	157.5	55.0	62.0	51.8	48.2	84.6	55.4	136.4	9.93	0.020
Al_5_Li_2_Co	Present	144.9	76.5	35.9	34.0	15.6	58.2	69.1	32.5	84.3	4.41	1.534
Al_5_Li_2_Cu	Present	134.6	122.0	37.3	41.8	39.3	36.6	68.4	41.6	103.8	7.03	0.055
Al_5_Li_2_Zn	Present	123.2	116.4	33.5	36.4	37.0	30.9	62.2	38.0	94.7	6.42	0.082
Al_6_LiZr	Present	153.9	143.0	55.7	60.5	51.6	47.2	82.4	54.6	134.2	9.88	0.026
Al_6_LiNb	Present	162.0	156.2	54.2	64.0	56.4	61.9	93.4	54.2	136.3	8.88	0.047
Al_6_LiMo	Present	90.3	146.3	39.6	50.9	86.8	67.9	85.0	20.2	56.1	1.48	17.33

## References

[1] Ning J., Zhang L.J., Bai Q.L., Yin X.Q., Niu J., Zhang J.X. (2017). Comparison of the microstructure and mechanical performance of 2A97 Al-Li alloy joints between autogenous and non-autogenous laser welding. Mater. Des..

[2] Bairwa M.L., Date P.P. (2004). Effect of heat treatment on the tensile properties of Al-Li alloys. J. Mater. Process. Technol..

[3] Ovri H., Lilleodden E.T. (2015). New insights into plastic instability in precipitation strengthened Al-Li alloys. Acta Mater..

[4] Rösner H., Kalogeridis A., Liu W., Pesicka J., Nembach E. (1997). Dislocation mechanisms in Al-rich Al-Li alloys. Mater. Sci. Eng. A.

[5] Flower H., Gregson P. (1987). Solid state phase transformations in aluminium alloys containing lithium. Mater. Sci. Technol..

[6] Williams D., Edington J. (1975). The precipitation of δ′(Al_3_Li) in dilute aluminium–lithium alloys. Met. Sci..

[7] Laverock J., Dugdale S.B., Alam M.A., Roussenova M.V., Wensley J.R., Kwiatkowska J., Shiotani N. (2010). Fermi surface of an important nanosized metastable phase: Al_3_Li. Phys. Rev. Lett..

[8] Pletcher B.A., Wang K.G., Glicksman M.E. (2012). Experimental, computational and theoretical studies of δ′ phase coarsening in Al-Li alloys. Acta Mater..

[9] Mogucheva A., Kaibyshev R. (2016). Microstructure and Mechanical Properties of an Al-Li-Mg-Sc-Zr Alloy Subjected to ECAP. Metals.

[10] Chabala J.M., Levi-Setti R., Soni K.K., Williams D.B., Newbury D.E. (1991). Secondary ion imaging of the distribution of δ′ (Al_3_Li) in Al-Li alloys. Appl. Surf. Sci..

[11] Gu B.P., Liedl G.L., Kulwicki J.H., Sanders T.H. (1985). Coarsening of δ′ (Al_3_Li) precipitates in an Al-2.8Li0.3Mn alloy. Mater. Sci. Eng..

[12] Hoyt J.J., Spooner S. (1991). The surface energy of metastable Al3Li precipitates from coarsening kinetics. Acta Metall. Et Mater..

[13] Lee B.C., Park J.K. (1998). Effect of the addition of Ag on the strengthening of Al_3_Li phase in Al-Li single crystals. Acta Mater..

[14] Mao Z., Seidman D.N., Wolverton C. (2016). The effect of vibrational entropy on the solubility and stability of ordered Al_3_Li phases in Al-Li alloys. APL Mater..

[15] Poduri R., Chen L.Q. (1998). Computer simulation of morphological evolution and coarsening kinetics of δ′ (Al_3_Li) precipitates in Al-Li alloys. Acta Mater..

[16] Li Z., Tse J.S. (2000). Ab initio studies on the vibrational and thermal properties of Al_3_Li. Phys. Rev. B.

[17] Yu H., Duan X., Ma Y., Zeng M. (2012). First Principles Study of Al-Li Intermetallic Compounds. Chin. J. Chem. Phys..

[18] Sluiter M., De F.D., Guo X.Q., Podloucky R., Freeman A.J. (1990). First-principles calculation of phase equilibria in the aluminum lithium system. Phys. Rev. B.

[19] Hu W.C., Liu Y., Li D.J., Zeng X.Q., Xu C.S. (2013). Mechanical and thermodynamic properties of Al_3_Sc and Al_3_Li precipitates in Al-Li-Sc alloys from first-principles calculations. Phys. B Condens. Matter.

[20] Wolverton C., Ozoliņš V. (2006). First-principles aluminum database: Energetics of binary Al alloys and compounds. Phys. Rev. B.

[21] Guo X., Podloucky R., Xu J., Freeman A.J. (1990). Cohesive, electronic, and structural properties of Al_3_Li: An important metastable phase. Phys. Rev. B.

[22] Yao J., Zhang C., Jiang Y., Tao H., Yin D. (2015). Prediction on elastic properties of off-stoichiometric L1_2_-Al_3_Li intermetallic due to point defects. Comput. Mater. Sci..

[23] Makineni S.K., Sugathan S., Meher S., Banerjee R., Bhattacharya S., Kumar S., Chattopadhyay K. (2017). Enhancing elevated temperature strength of copper containing aluminium alloys by forming L1_2_ Al_3_Zr precipitates and nucleating θ″ precipitates on them. Sci. Rep..

[24] Hu H., Wu X., Wang R., Jia Z., Li W., Liu Q. (2016). Structural stability, mechanical properties and stacking fault energies of TiAl_3_ alloyed with Zn, Cu, Ag: First-principles study. J. Alloys Compd..

[25] Gu J., Bai J., Zhu Y., Qin Y., Gu H., Zhai Y., Ma P. (2016). First-principles study of the influence of doping elements on phase stability, crystal and electronic structure of Al_2_Cu (θ) phase. Comput. Mater. Sci..

[26] Kubouchi M., Hayashi K., Miyazaki Y. (2016). Electronic structure and thermoelectric properties of boron doped Mg_2_Si. Scr. Mater..

[27] Segall M., Lindan P.J., Probert M.A., Pickard C., Hasnip P., Clark S., Payne M. (2002). First-principles simulation: Ideas, illustrations and the CASTEP code. J. Phys. Condens. Matter.

[28] Perdew J.P. (1986). Density-functional approximation for the correlation energy of the inhomogeneous electron gas. Phys. Rev. B.

[29] Vanderbilt D. (1990). Soft self-consistent pseudopotentials in a generalized eigenvalue formalism. Phys. Rev. B.

[30] Perdew J.P., Burke K., Ernzerhof M. (1996). Generalized Gradient Approximation Made Simple. Phys. Rev. Lett..

[31] Cohen A.J., Mori-Sánchez P., Yang W. (2008). Insights into current limitations of density functional theory. Science.

[32] Yoshiyama T., Hasebe K., Mannami M. (2007). Al_3_Li Superlattice in Al-4.5 wt % Li Alloy. J. Phys. Soc. Jpn..

[33] Sahu B.R. (1995). Electronic structure and bonding of ultralight LiMg. Mater. Sci. Eng. B.

[34] Wang J.-H., Lu Y., Zhang X.-L., Shao X.-H. (2018). The elastic behaviors and theoretical tensile strength of γ-TiAl alloy from the first principles calculations. Intermetallics.

[35] Mouhat F., Coudert F.-X. (2014). Necessary and sufficient elastic stability conditions in various crystal systems. Phys. Rev. B.

[36] Tian J., Zhao Y., Wang B., Hou H., Zhang Y. (2018). The structural, mechanical and thermodynamic properties of Ti-B compounds under the influence of temperature and pressure: First-principles study. Mater. Chem. Phys..

[37] Xiao B., Feng J., Zhou C.T., Jiang Y.H., Zhou R. (2011). Mechanical properties and chemical bonding characteristics of Cr_7_C_3_ type multicomponent carbides. J. Appl. Phys..

[38] Qi Y.Y., Mu Y., Cheng Y., Ji G.F. (2015). Pressure effect on electronic, elastic and optical properties of Eu:CaF_2_ crystal: A first-principles study. Philos. Mag..

[39] Mao Z., Chen W., Seidman D.N., Wolverton C. (2011). First-principles study of the nucleation and stability of ordered precipitates in ternary Al-Sc-Li alloys. Acta Mater..

[40] Hill R. (1952). The elastic behaviour of a crystalline aggregate. Proc. Phys. Soc..

[41] Watt J.P., Peselnick L. (1980). Clarification of the Hashin-Shtrikman bounds on the effective elastic moduli of polycrystals with hexagonal, trigonal, and tetragonal symmetries. J. Appl. Phys..

[42] Gao F.M., Gao L.H. (2010). Microscopic models of hardness. J. Superhard Mater..

[43] Chen X.Q., Niu H., Li D., Li Y. (2011). Modeling hardness of polycrystalline materials and bulk metallic glasses. Intermetallics.

[44] Yousef E.S., El-Adawy A., El-Kheshkhany N. (2006). Effect of rare earth (Pr_2_O_3_, Nd_2_O_3_, Sm_2_O_3_, Eu_2_O_3_, Gd_2_O_3_ and Er_2_O_3_) on the acoustic properties of glass belonging to bismuth–borate system. Solid State Commun..

[45] Zhang W., Chai C., Song Y., Fan Q., Yang Y. (2018). Structural, Mechanical, Anisotropic, and Thermal Properties of AlAs in oC12 and hP6 Phases under Pressure. Materials.

[46] Zhang H., Shang S., Wang Y., Saengdeejing A., Chen L., Liu Z. (2010). First-principles calculations of the elastic, phonon and thermodynamic properties of Al_12_Mg_17_. Acta Mater..

[47] Nong Z.-S., Zhu J.-C., Yang X.-W., Cao Y., Lai Z.-H., Liu Y., Sun W. (2014). First-principles calculations of the stability and hydrogen storage behavior of C14 Laves phase compound TiCrMn. Solid State Sci..

[48] Haines J., Leger J., Bocquillon G. (2001). Synthesis and design of superhard materials. Ann. Rev. Mater. Res..

[49] Wang Y., Yang J., Huang J., Wang W., Ye Z., Chen S., Zhao Y. (2018). First-principles calculations on physical properties of Ni_3_Snx binary system intermetallic compounds and Ni/Ni3Sn interfaces in Nickel-Tin TLPS bonding layer. Intermetallics.

[50] Ledbetter H., Migliori A. (2006). A general elastic-anisotropy measure. J. Appl. Phys..

[51] Ranganathan S.I., Ostoja-Starzewski M. (2008). Universal elastic anisotropy index. Phys. Rev. Lett..

[52] Hu H., Wu X., Wang R., Li W., Liu Q. (2016). Phase stability, mechanical properties and electronic structure of TiAl alloying with W, Mo, Sc and Yb: First-principles study. J. Alloys Compd..

[53] Ravindran P., Fast L., Korzhavyi P.A., Johansson B., Wills J., Eriksson O. (1998). Density functional theory for calculation of elastic properties of orthorhombic crystals: Application to TiSi_2_. J. Appl. Phys..

